# Quality assurance of radiotherapy in the ongoing EORTC 1420 “Best of” trial for early stage oropharyngeal, supraglottic and hypopharyngeal carcinoma: results of the benchmark case procedure

**DOI:** 10.1186/s13014-021-01809-2

**Published:** 2021-05-01

**Authors:** J-J Stelmes, E. Vu, V. Grégoire, C. Simon, E. Clementel, J. Kazmierska, W. Grant, M. Ozsahin, M. Tomsej, L. Vieillevigne, C. Fortpied, E. C. Hurkmans, A. Branquinho, N. Andratschke, F. Zimmermann, D.-C. Weber

**Affiliations:** 1 Radiation Oncology Department, Oncology Institute of Southern Switzerland, Via Athos Gallino 12, 6500 Bellinzona, Switzerland; 2 Department of Radiation Oncology, Kantonsspital St. Gallen, St. Gallen, Switzerland; 3 Lyon University Hospital, Lyon, France; 4 Lausanne University Hospital, Lausanne, Switzerland; 5 EORTC Headquarters, Brussels, Belgium; 6 Greater Poland Cancer Center, Poznan, Poland; 7 Gloucestershire Hospitals, NHS Foundation Trust, Gloucester, UK; 8 Hospital of Charleroi, Charleroi, Belgium; 9 Institut Claudius Regaud IUCT, Toulouse, France; 10 Catharina Hospital, Eindhoven, The Netherlands; 11 Centro Hospitalar Lisboa Norte, Lisbon, Portugal; 12 University Hospital Zurich, Zurich, Switzerland; 13 University Hospital of Basel, Basel, Switzerland; 14 University Hospital of Bern, Bern, Switzerland; 15 Paul-Scherrer-Institute, Villigen, Switzerland

**Keywords:** EORTC 1420 Best-of trial, Swallowing, Organs at risk, Benchmark case

## Abstract

**Introduction:**

The current phase III EORTC 1420 Best-of trial (NCT02984410) compares the swallowing function after transoral surgery versus intensity modulated radiotherapy (RT) in patients with early-stage carcinoma of the oropharynx, supraglottis and hypopharynx. We report the analysis of the Benchmark Case (BC) procedures before patient recruitment with special attention to dysphagia/aspiration related structures (DARS).

**Materials and methods:**

Submitted RT volumes and plans from participating centers were analyzed and compared against the gold-standard expert delineations and dose distributions. Descriptive analysis of protocol deviations was conducted. Mean Sorensen-Dice similarity index (mDSI) and Hausdorff distance (mHD) were applied to evaluate the inter-observer variability (IOV).

**Results:**

65% (23/35) of the institutions needed more than one submission to achieve Quality assurance (RTQA) clearance. OAR volume delineations were the cause for rejection in 53% (40/76) of cases. IOV could be improved in 5 out of 12 OARs by more than 10 mm after resubmission (mHD). Despite this, final IOV for critical OARs in delineation remained significant among DARS by choosing an aleatory threshold of 0.7 (mDSI) and 15 mm (mHD).

**Conclusions:**

This is to our knowledge the largest BC analysis among Head and neck RTQA programs performed in the framework of a prospective trial. Benchmarking identified non-common OARs and target delineations errors as the main source of deviations and IOV could be reduced in a significant number of cases after this process. Due to the substantial resources involved with benchmarking, future benchmark analyses should assess fully the impact on patients’ clinical outcome.

**Supplementary Information:**

The online version contains supplementary material available at 10.1186/s13014-021-01809-2.

## Highlights

First BC analysis evaluating delineation of swallowing organs, planning volume, and planning variations.

## Introduction

Due to the emergence of HPV related oropharyngeal cancer and the increase in survival over the past decades, quality of life and in particular preservation of swallowing function becomes of paramount importance [1, 2]. Thus, novel strategies such as intensity modulated radiation therapy (IMRT) with defined constraints to dysphagia/aspiration related structures (DARS) and trans-oral surgery have been developed to provide better functional outcome whilst preserving treatment efficacy [3, 4]. A recent randomized phase III trial evaluated a dysphagia optimized intensity modulated radiotherapy (Do-IMRT) in comparison to a standard IMRT approach: The authors could show that a significantly reduced dose to the pharyngeal constrictor muscles (PCM) lead to an improvement in MDADI scores [5]. However, there is a lack of evidence to guide the choice between these two treatment options. The EORTC 1420 Best-of trial (NCT02984410) has been developed to specifically answer the question of functional equivalence between these two treatments [6]. The primary endpoint is the patient reported swallowing function over the first year evaluated by the MD Anderson Dysphagia Inventory [7].

As aberrations of the radiotherapy (RT) protocols can influence trial results [8, 9] all EORTC trials involving RT, have a dedicated trial specific Quality Assurance (RTQA) program [10,11,12] in order to increase validation of the results [13, 14].

Thus, as part of such a program, participating institutions are required to submit a benchmark case (BC) prior to patient enrollment. Benchmarking assesses sources of variation in delineation and treatment planning and ensures that the specific delineation and dose/volume guidelines for a given trial are correctly understood.

The BC in this trial is based on a selected case which fulfills the inclusion criteria of the trial. The RT volumes and corresponding reference plan have been created by experts in the field of Head and neck cancer (HNC) and compared to the single institution (RT volumes and RT plan). As swallowing function is the primary endpoint, special attention is required in delineating the DARS in reference to published guidelines [15].

The aim of this study was to investigate the performances of the participating institutions in the trial with respect to RT planning and assess the inter-observer variability (IOV). Finally, we aimed to evaluate the effectiveness of the BC procedure in reducing IOV across participating institutions. To our knowledge, this is the largest benchmark case evaluation of a currently ongoing phase III HNC trial with a specific dedication to DARS variability.

## Materials and methods

A case summary including MRI screenshots were sent to the institutions. The detailed case description can be found as supplemental material. The first step of the exercise consisted of contouring all requested structures of the protocol and sending them back for revision. After volume approval, the institutions were invited to create a treatment plan according to protocol instructions (Chapter 4.5). Three radiation oncologists (J.K; W.G; M.O) specialized in head and neck cancer (ROSHNC) as two medical physicists (M.T; L.V) were previously elected to assess these submissions. Vodca RT software package (Visualization and Organization of Data for Cancer Analysis, Medical Software Solutions GmbH; Hagendorm Switzerland) was used for review.

The RTQA experts verified each investigator delineation and dose planning against a previously defined gold standard (GS). In this analysis, GS was made based on the following process: First, contouring of the BC was done separately by each ROSHNC based on pre-defined guidelines [15]. In a second step, a comparison was made, and eventual discrepancies discussed during a dedicated meeting. This process was repeated until each reviewer agreed on every single volume.

Volume definitions and dose prescriptions:

The therapeutic volume included the primary tumor and in case of N1 disease the positive lymph node. The precise contouring instructions including margins were based on current international guidelines [16]. The prophylactic volume included all nodal areas at risk for microscopic infiltration in addition to the therapeutic volume. Selection of elective lymph node stations were based on the publication by Biau et al. [17]. Unilateral irradiation was requested in case the tumor was more than 1 cm away from midline. IMRT with a simultaneous integrated boost (SIB) was mandatory. Sixty-six and or 70 Gy (T2 tumor and N1-node) in 2 Gy per fraction with 6 fractions/week was applied to the therapeutic target volumes. The elective volumes received a total dose of 54.45 Gy (T1 Tumor) and 56 Gy (T2 and N1-node) respectively. Furthermore, also for organ at risks, clear instructions on delineation were specified in the protocol [15].

Per protocol every criterion was classified in acceptable or unacceptable variation according the Global Harmonization Group Guidelines [18, 19]. Concise feedback was given and in case of unacceptable deviations, a resubmission with the needed changes was requested. If all parameters were protocol conformal or within guidelines-defined limits of acceptability, the benchmark procedure was considered completed. A summary of the predefined review criteria can be found in the supplementary material.

Review results by the RTQA experts were collected in an Excel spreadsheet and summary statistics about global RTQA performance was calculated. Furthermore, in order to evaluate IOV, the mean Sørensen-Dice Similarity Index (mDSI) and the mean percentile Hausdorff distance (mHD) were calculated retrospectively using MIM Software Inc, Cleveland, Ohio, USA, Version 6.9.2.The mDSI, also called the overlap index, is based upon the formula:

$$DSI=2\times \frac{A\cap B}{A\cup B}$$

with A and B representing the volumes of the contoured region of interest performed by the expert and one of the institutions, respectively. Values close to 1 indicate similar contours [20]. The HD measures the largest minimal distance between two boundaries. The ideal case with perfect superimposition corresponds to a HD equal to zero. Means and standard deviations were calculated for Dice and HD indexes and means and ranges for doses to OARs. Descriptive statistics and plots were generated using SAS software (SAS Institute Inc., Cary, NC, USA) [21].

## Results

Thirty-five centers have participated in the EORTC 1420 BC analysis. Sixty-five percent (23/35) of them needed more than one submission (2, 1–4). In total, the RTQA intervention has detected 76 unacceptable deviations. Among them, OAR delineations were the cause for rejection in 53% (40/76) followed by inadequate target delineations in 35% (27/76). Lastly, concerning planning deviations, non-fulfillment of the given dose-constraints was notified in 12% (9/76). However, at a single evaluation, we found that elective volume delineation (prophylactic PTV), resulted as the major cause of rejection in 24% (19/76). Among OARs, pharyngeal constrictor muscles (PCM) (8/76) were the most frequent organs. A detailed table can be found as supplementary material. mDSI/mHD indexes between initially rejected and finally accepted volumes have been investigated: Concerning mDSI, the differences between the first and last submissions were marginal with a mean difference ranging from − 0.01 to 0.20 across all volumes including prophylactic PTV (pPTV) (mDSI difference of 0.07). On the other hand, the mean difference in mHD has noteworthy improved for almost all RT volumes and were as large as − 13.1 mm for the mandible (M) and − 10.8 mm for the extended oral cavity (EOC). More precisely, in 5 out of 12 volumes, the mHD decreased by even more than 10 mm including for prophylactic PTV (mHD change: 10.6) (Table 1). In addition, a proportional correlation between major rejection rate and the subsequent improvement in terms of mHD seems plausible. Indeed, pPTV and PCM, both figured among the most frequent causes of BC rejection and had their mHD improved by more than 10 mm.

**Table 1:** Comparison of mDSI/mHD index evaluation for initially rejected and finally accepted volumes

Index	mDSI (SD)			mHD (SD)		
Volumes(n = 67)	First	Final	Difference	First	Final	Difference
Planning organ-at-risk volume of the brainstem (3)	0.74 (0.16)	0.75 (0.14)	0.01 (0.02)	8.94 (4.26)	7.88 (3.38)	− 1.1 (1.1)
Cervical esophagus (4)	0.49 (0.10)	0.33 (0.16)	− 0.16 (0.13)	27.22 (11.98)	18.83 (8.44)	− 8.4 (14.0)
Cricoid pharyngeal inlet (5)	0.36 (0.22)	0.38 (0.09)	0.03 (0.20)	13.42 (6.75)	13.19 (3.87)	− 0.2 (9.6)
Contralateral sub-mandibular gland (1)	0.83 (.)	0.83(.)	0.00(.)	5.37 (.)	5.37 (.)	0.0 (.)
Extended oral cavity—PTV (5)	0.76 (0.02)	0.84 (0.08)	0.08 (0.06)	26.36 (4.22)	15.56 (9.19)	− 10.8 (9.7)
Glottis (3)	0.43 (0.19)	0.56 (0.15)	0.14 (0.34)	13.28 (7.63)	8.58 (4.89)	− 4.7 (12.3)
Mandible (6)	0.88 (0.04)	0.91 (0.02)	0.03 (0.03)	19.84 (8.59)	6.75 (4.98)	− 13.1 (8.8)
Pharyngeal constrictor muscles (8)	0.47 (0.16)	0.59 (0.09)	0.12 (0.12)	30.34 (8.83)	19.70 (3.65)	− 10.6 (8.4)
Prophylactic PTV (19)	0.70 (0.13)	0.78 (0.05)	0.07 (0.13)	26.23 (16.33)	15.64 (4.59)	− 10.6 (17.1)
Therapeutic PTV (8)	0.64 (0.15)	0.70 (0.10)	0.06 (0.11)	11.14 (4.05)	10.17 (2.31)	− 1.0 (3.7)
Planning organ-at-risk volume of the spinal cord (3)	0.78 (0.07)	0.80 (0.09)	0.01 (0.02)	6.61 (2.73)	6.68 (2.80)	0.1 (1.1)
Supraglottic larynx (2)	0.42 (0.31)	0.41 (0.29)	− .01 (0.02)	21.03 (18.35)	23.33 (15.09)	2.3 (3.3)

**Fig. 1:**
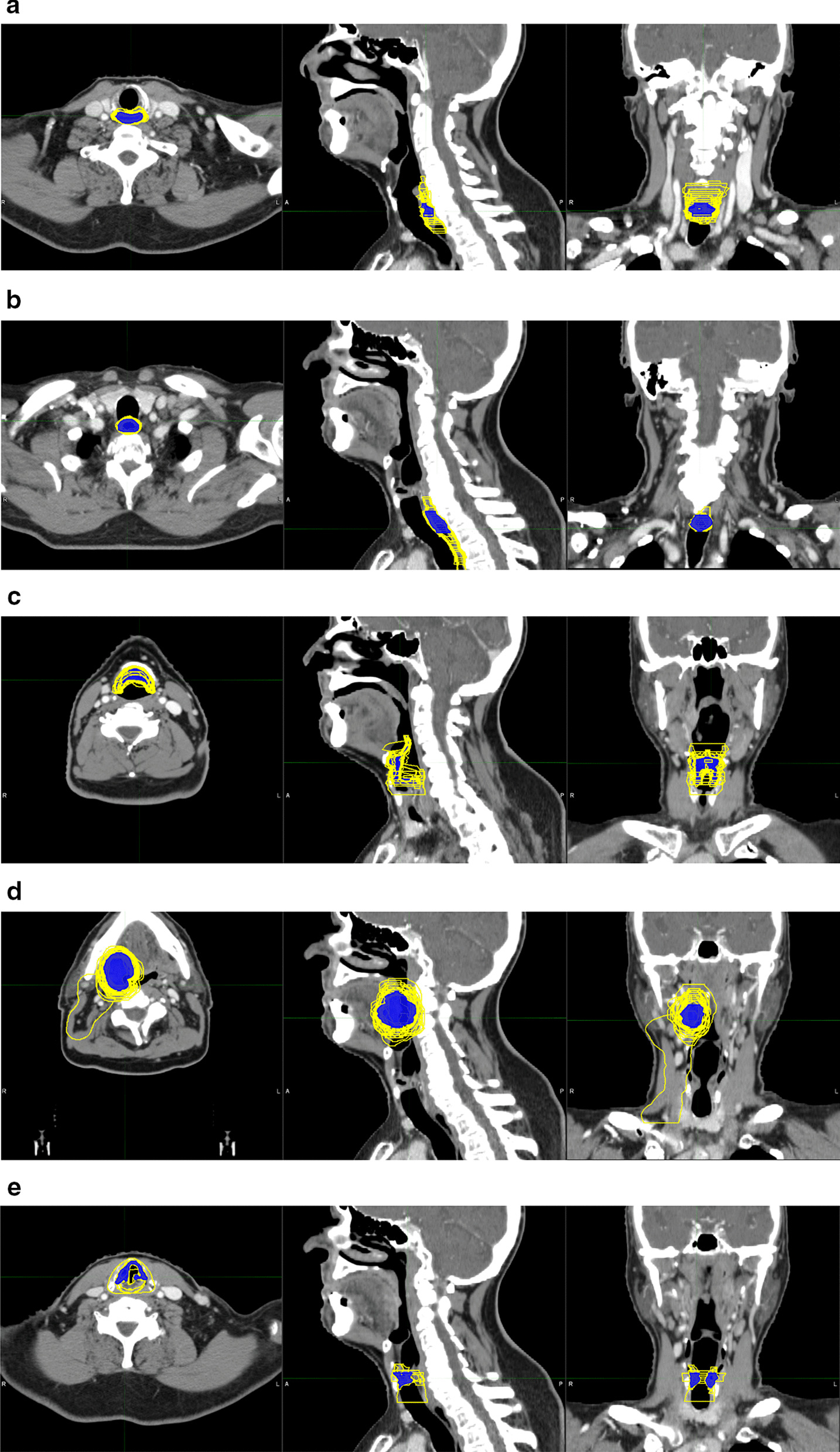
Graphic representation on axial, sagittal and coronal planes of interobserver variation between institutions concerning the cricopharyngeal inlet (**a**), cervical esophagus (**b**), supraglottic larynx (**c**), therapeutic PTV (**d**) and glottis (**e**). The reference delineation is represented in blue outline

**Fig. 2:**
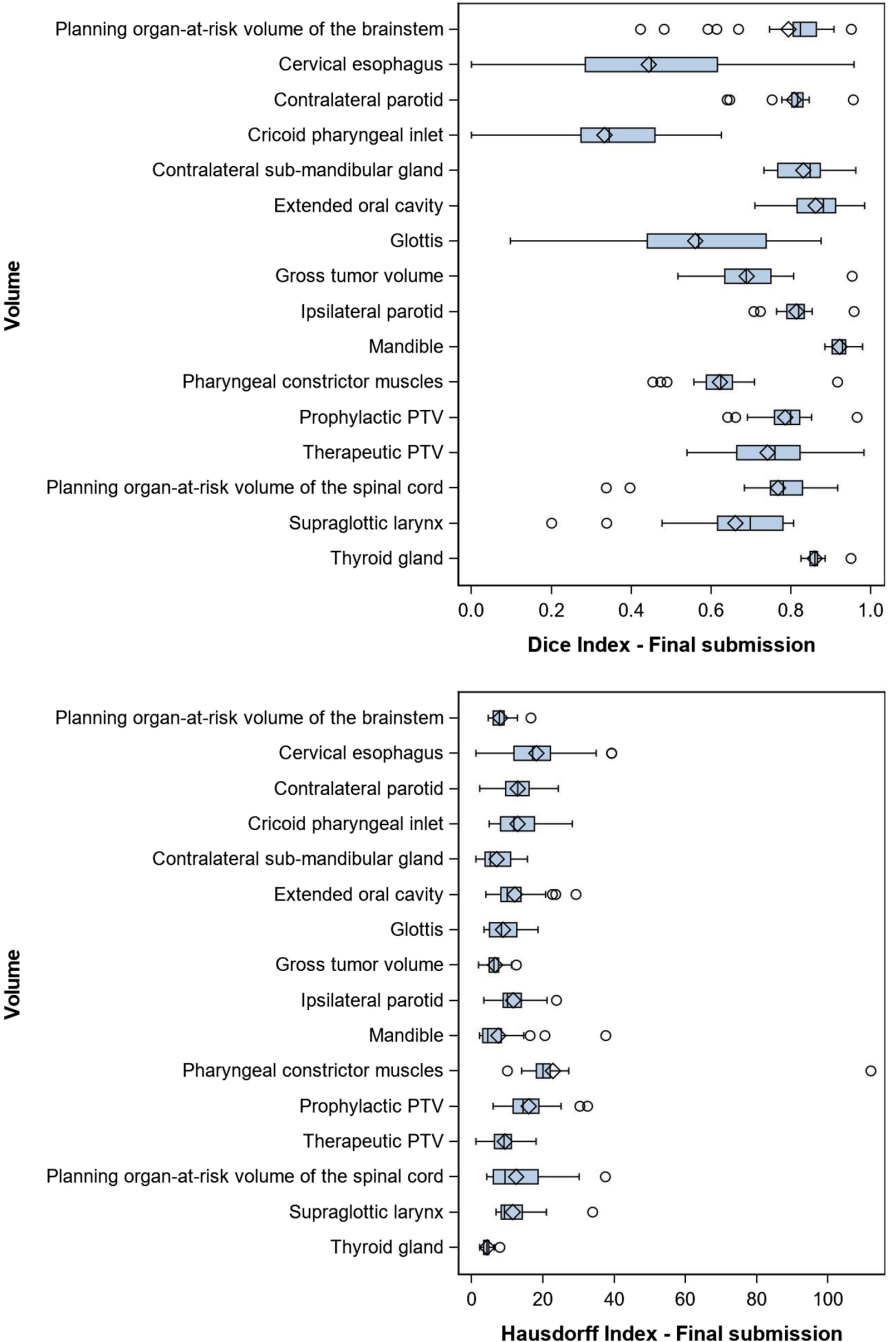
Boxplots of the Dice conformity Index and Hausdorff Index for each RT volume at final submission

Globally, all volumes confound, final IOV varied between 0.33–0.92 and 4.36–22.86 for mDSI and mHD (Table 2). Regarding each volume separately, the lowest agreement in confront to expert delineations based on the mDSI was observed for the cricopharyngeal inlet (CPI), the cervical esophagus (CE) and the glottis (G) with a mean ± SD of 0.33 ± 0.16, 0.44 ± 0.22 and 0.56 ± 0.20 respectively. Figure 1 shows a visual representation of the different contours made by the centers (yellow) in comparison with the expert delineation (blue). At the same time, based on the mHD index comparison, the lowest agreements were found for the PCM, CE, and prophylactic PTV with a mHD ± SD of 22.9 ± 17.28, 18.20 ± 4.2 and 16.05 ± 6.03. The organs who showed the highest variation in terms of SD, across all institutions at final submissions for the mDSI are the CE, G and supraglottic larynx (SL) with a SD of around 0.2 and for the mHD: PCM (17.28), CE (9.64) and the Spinal cord PRV (8.79). Figure 2 displays the box plots of the mDSI and mHD indexes for each volume at the final submission.

**Table 2:** Mean DSI and HD indexes for the final volumes

Final volume	DSI (SD)	HD (SD)
Planning organ-at-risk volume of the brainstem	0.79 (0.12)	8.05 (2.59)
Cervical esophagus	0.44 (0.22)	18.20 (9.64)
Contralateral parotid	0.81 (0.05)	12.94 (5.27)
Cricoid pharyngeal inlet	0.33 (0.16)	12.93 (6.12)
Contralateral sub-mandibular gland	0.83 (0.06)	7.02 (4.28)
Extended oral cavity-PTV	0.86 (0.07)	12.14 (6.13)
Glottis	0.56 (0.20)	8.81 (4.28)
Ipsilateral parotid	0.81 (0.04)	11.71 (4.24)
Mandible	0.92 (0.02)	7.52 (7.47)
Pharyngeal constrictor muscles	0.62 (0.08)	22.86 (17.28)
Prophylactic PTV	0.79 (0.06)	16.05 (6.03)
Therapeutic PTV	0.74 (0.10)	9.35 (3.47)
Planning organ-at-risk volume of the spinal cord	0.77 (0.13)	12.52 (8.79)
Supraglottic larynx	0.66 (0.16)	11.58 (6.05)
Thyroid gland	0.86 (0.02)	4.36 (1.20)

**Fig. 3:**
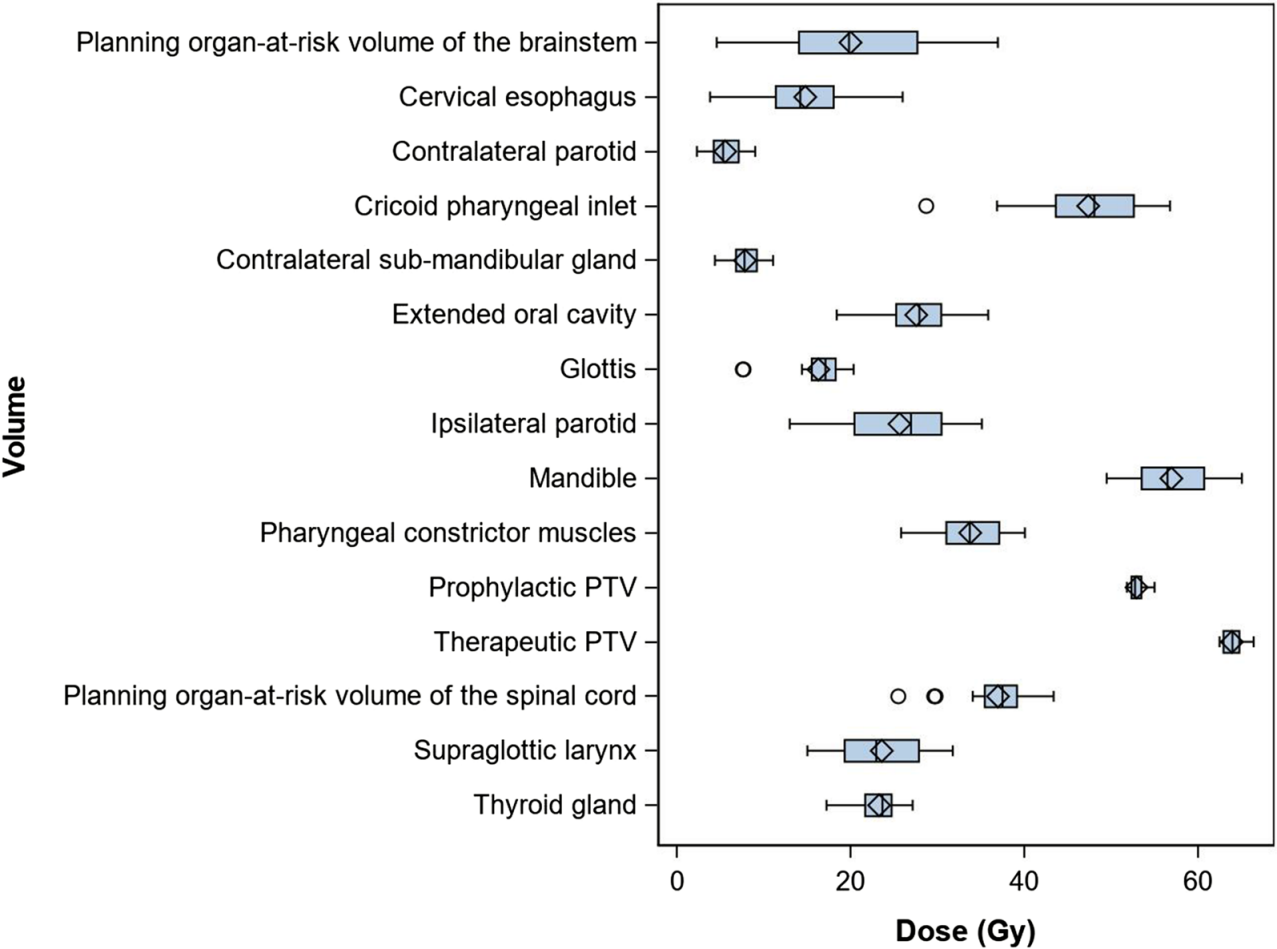
Dose variations received by RT volumes based on the protocol dose contraints

At the final evaluation all dose constraints of the EORTC 1420 protocol were fulfilled without greater difficulty (Table 3). The organ at risk which was closest to the given dose requirements was the thyroid gland with a Dmean of 23 Gy (Protocol: Dmean < 25 Gy). The brainstem PRV was the most variable structure in terms of dose distribution with a mean D_max_ of 20 Gy (range: 4.6–37 Gy) followed by the EOC with a D_mean_ of 27.5 (range: 18.4–35.8 Gy). Figure 3 shows the Dose variations regarding each RT volume.

**Table 3:** Mean institutional dose distributions and dose requirements of the EORTC 1420 protocol for the different volumes

Volumes	Mean institutional doses (SD)	Dose requirements given by the EORTC 1420 Protocol
Planning organ-at-risk volume of the brainstem	Dmax = 19.96 (9.12)	Dmax < 35 Gy
Cervical esophagus	Dmean = 14.75 (5.43)	Dmean < 30 Gy
Contralateral parotid	Dmean = 5.53 (1.79)	Dmean < 5–10 Gy
Cricoid pharyngeal inlet	D2% = 47.34 (6.98)	D2% < 66 Gy
Contralateral sub-mandibular gland	Dmean = 7.82 (1.67)	Dmean < 10 Gy
Extended oral cavity-PTV	Dmean = 27.54 (4.66)	Dmean < 30–35 Gy
Glottis	Dmean = 16.26 (3.56)	Dmean < 20 Gy
Ipsilateral parotid	Dmean = 25.60 (5.83)	Dmean < 30–35 Gy
Mandible	D2% = 56.92 (4.59)	D2% < 66 Gy
Pharyngeal constrictor muscles	Dmean = 33.75 (3.95)	Dmean < 30–40 Gy
Prophylactic PTV	D95% = 52.91 (0.81)	D95% > 51.7 Gy
Therapeutic PTV	D95% = 63.89 Gy (1.04)	D95% > 62.7 Gy
Planning organ-at-risk volume of the spinal cord	Dmax = 36.95 (4.05)	Dmax < 40 Gy
Supraglottic larynx	Dmean = 23.56 (4.90)	Dmean < 45–50 Gy
Thyroid gland	Dmean = 23.26 (2.05)	Dmean < 25 Gy

## Discussion

Thirty-five European institutions with in total 81 submitted benchmarks participated in this ancillary analysis inside an ongoing EORTC Head and neck trial. Hence, to our knowledge this represents the largest BC analysis in current literature among Head and neck quality assurance programs. This analysis followed the suggestions on reporting items for studies on IOV in volume delineation made by Vinod et al. [22].

First, we could affirm that the major source of protocol variations relies on the delineation process, be it for OARs (53%) or target volumes (35%). This can be simply explained by a greater number of non-routing DARS required by the protocol. Considering each volume separately, pPTV was by far the major cause of rejection (24%). Several reasons could be identified as for example non protocol conformal selection of prophylactic lymph node regions, inadequate boundaries and inclusion of natural barriers inside the clinical target volume (CTV). These results are in par with the two other BC analysis inside the EORTC Head and neck Trials portfolio: In the EORTC 22071-26071 BC analysis, a trial evaluating the benefit of Panitumumab in addition to adjuvant chemoradiotherapy in locally advanced resected HNC revealed that 43.5% of the 23 submitted cases presented major protocol deviation on target volume delineations (GTVs, CTVs and PTVs) [11]. Additionally, Christiaens et al. published the EORTC 1219-Dahanca-29 BC analysis, an intergroup trial evaluating the influence of Nimorazole in patients with locally advanced head and neck cancer [23]. This work was done on 34 delineation BCs submitted by 19 centers. The authors reported a similar rejection rate of 63% (65% in our analysis). Furthermore, incorrect selection of prophylactic lymph node regions, were the major cause of rejection. This highlights the fact that elective nodal irradiation in terms of selection of LN regions and their corresponding delimitations still represents a major source of heterogeneity despite current guidelines. With regard to OAR delineations, in the EORTC 1219 BC trial, absence of contours represented the major reason for first refusal (10%). In our study, PCM was the principal reason for rejection. Only a minority (12%) of variations were due to planning issues.

After RTQA intervention, no notable difference could be detected in terms of mDSI. In the opposite, regarding mHD, an improvement in almost all volumes was observed. More precisely, in 5 out of 12 volumes, the mHD difference was even more than 10 mm. In the EORTC 1219 trial an improvement from the first to final submission in mDSI and mHD values was found to be increased for the prophylactic CTV (mDSI: 0.09 mHD: − 11.6). On the other hand, the therapeutic CTV volume was only marginally improved (mDSI: 0.01; mHD: 0.4 mm). These findings were in par with our results highlighting an improvement for the prophylactic target volume of—10.6 (SD 17) in mHD and 0.07 in mDSI. Identically, the changes in therapeutic volume metrics were negligible. Furthermore, regarding the PCM, mHD increased by—10.6 (SD 17). Their seems to be a relationship between the rejected OARs and IOV improvement. In particular prophylactic PTV and pharyngeal constrictor muscles, both figured among the most frequent causes of BC rejection and had their mHD improved by more than 10 mm. In the counterpart, therapeutic PTV improved after RTQA intervention but with a smaller margin even if represented by a relatively high rejection rate. This is probably because changes in volume resubmissions are smaller.

The apparent discrepancy between both indexes relies on their different nature: The Dice coefficient is a volume-based metric and therefore commonly used to measure reproducibility. In the opposite, mHD index is part of the spatial distance-based metrics and consequently makes it especially interesting for highlighting the largest mismatch between the consensus contours and the submitted contours [24]. Therefore mHD is often more sensitive for benchmarking, simply by the fact that most critical sources of fluctuance, from an anatomical point of view derive from the upper and lower anatomical boundaries [25]. This criterion is most critical for longitudinal and narrow organs at risk as for example the CE and PCM and therefore mHD index is best fit to evaluate such discrepancies.

In addition, final mDSI and mHD metrics varied between 0.33 (cricopharyngeal inlet)—0.92 (mandible) and 22.86 (pharyngeal constrictor muscles)—4.36 (thyroid gland) respectively. By choosing an aleatory threshold of 0.7 for mDSI and 15 mm for mHD, the CE-CPI-PCM-SL and glottis are in the high risk IOV group for mDSI variability as the PCM-CE and prophylactic PTV are in the high IOV group for mHD variability. Furthermore, after excluding one single outlier of PCM, CE had the highest dispersion around the mean for both metrics (mDSI: 0.22; mHD: 9.64). In conclusion, these outcomes reinforce our hypothesis that new non-routine OARs in addition to the elective lymph node stations are at highest risk for IOV. Another hypothesis is that, due to low experience in non-routine OAR contouring, participating institutions tend to overestimate the true size of the organs.

In addition, in the EORTC 1219 DAHANCA BC analysis, the highest disagreement was observed for the larynx (mDSI/mHD 0.46 and 32.9) and oral cavity (mDSI/mHD: 0.49 and 19.08). This difference is increased in confront to our findings [(supraglottic larynx: mDSI/mHD: 0.66 and 11.58) and (oral cavity: mDSI/mHD: 0.86 and 12.14)]. This difference can be mainly explained by the fact that clear guidelines on organ at risk contouring were absent at that time.

Regarding the potential impact of volume IOV on dose distribution, no such evaluation could be made in our dataset as plans were created only in case volume acceptability was granted by the reviewers. However, in the EORTC 22071-26071 trial, the authors showed no statistically significant correlation between the PTV volume and the ability to meet parotid dose constraints (*p* > 0.20), neither between parotid contour acceptability nor meeting constraints (*p* = 0.17). Therefore, we hypothesize a low impact of IOV on dosimetry variations in case protocol requirements are fulfilled. Evaluating the potential impact of volume IOV on clinical outcomes, a review of several BC analysis realized among EORTC trials could not reveal any correlation between protocol conformal benchmarking and clinical results for a given trial [26].

Concerning the dose evaluation, no major difficulties were notified in reaching the given dose-constraints. Brainstem PRV was the most variable OAR. This finding is unsurprising as the brainstem tolerance in this protocol is far below the clinically recognized tolerance. Therefore, the participating institutions made no effort to optimize the dose to the brainstem.

Some limitations of this study should be mentioned: More accurate methods have been introduced since to estimate and reduce IOV, as the STAPLE ( simultaneous truth and performance level estimation) algorithm [27]. This method is a validated formula which considers a collection of segmentations and estimates the ground truth based on the performance level by each segmentation. In addition, novel strategies for implementing OAR contouring guidelines at a national scale [28], visual atlases [29] and the use of digital platforms [30] have been proposed to further reduce IOV. Even more recently, artificial intelligence systems have been studied to simplify the workflow of radiotherapy [31]. In particular deep learning contouring (DLC) for Head and neck OARs were developed with satisfying preliminary results [32]. Indeed, DSI as mean and max dose difference were found to be significantly improved in 19 out of 22 OARs compared to the atlas based contouring (ABAS) by 0.15, 3.1 Gy and 3.3 Gy respectively[33]. The impact on dose distributions after Benchmark resubmissions could not be analyzed due to the internal process of planning once the volumes have been already been approved.

In conclusion, for this specific HNC trial with dysphagia as the primary endpoint and therefore critical relevance of correct delineation of not routinely delineated OARs, benchmarking was an effective tool to avoid protocol deviation and reduce IOV. On the other hand, in times of global increase of research costs, financing academic trials becomes a real challenge. We therefore recommend for future benchmark analyses an evaluation on the impact on dosimetry and clinical outcome as part of every trial initiation package [34].

## Supplementary Information

**Additional file 1:** BC description, protocol predefined criterions for BC review, and corresponding number of all unacceptable criterions.

## Data Availability

Data is available at EORTC HQ, Brussels, Belgium.

## Abbreviations

BC: Benchmark case
HPV: Human papilloma virus
IMRT: Intensity modulated radiation therapy
DARS: Dysphagia/aspiration related structures
MDADI: MD Anderson dysphagia inventory
RT: Radiotherapy
RTQA: Radiationtherapy quality assurance
ROSHNC: Radiationoncologist specialized in head and neck cancer
GS: Gold standard
SIB: Simultaneous integrated boost
mDSI: Mean Sorensen–Dice similarity index
mHD: Mean percentile Hausdorff distance
OAR: Organs at risk
PCM: Pharyngeal constrictor muscles
SD: Standard deviations
CPI: Cricopharyngeal inlet
CE: Cervical esophagus
G: Glottis
SL: Supraglottic larynx
PRV: Planning at risk volume
Dmean: Mean dose
IOV: Interobserver variability
CTV: Clinical target volume
pPTV: Prophylactic planning target volume
