# Supplementary material for: Quality assurance of radiotherapy in the ongoing EORTC 1420 “Best of” trial for early stage oropharyngeal, supraglottic and hypopharyngeal carcinoma: results of the benchmark case procedure
Source: Radiat Oncol. 2021 May 1;16:81. doi: 10.1186/s13014-021-01809-2 (PMC8088557; doi:10.1186/s13014-021-01809-2)
Supplement: Supplementary file 1 — Additional file 1: BC description, protocol predefined criterions for BC review, and corresponding number of all unacceptable criterions. [file 13014_2021_1809_MOESM1_ESM.docx]

**Case description:**

The case consisted of a 77-year old man with a symptomatic p16-negative squamous cell carcinoma of the right tonsillar fossa. The tumor did not infiltrate the soft palate, nor the glosso-tonsillar sulcus nor the para-pharyngeal space. Final staging was a cT1N0M0 (UICC 7^th^ edition). A T1 with gadolinium enhancement and a T2 sequence were attached as helping tools for delineation.


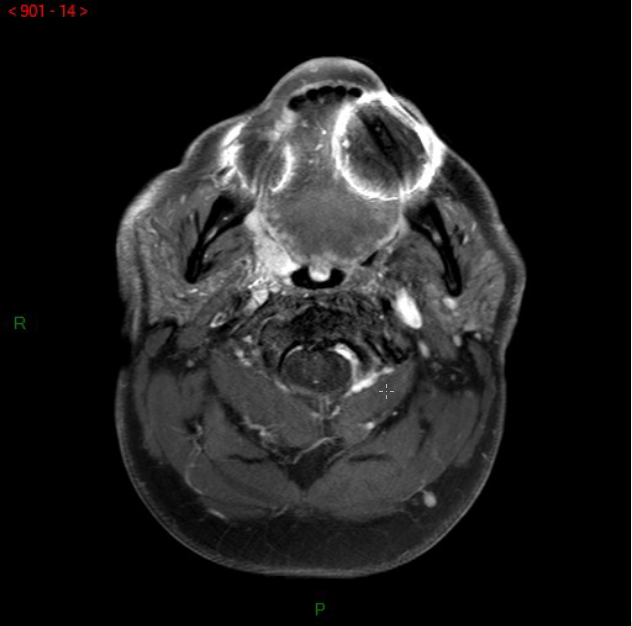

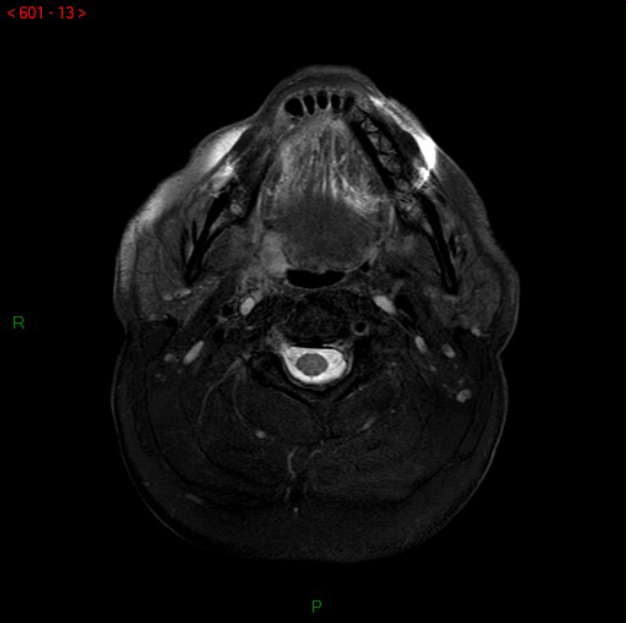


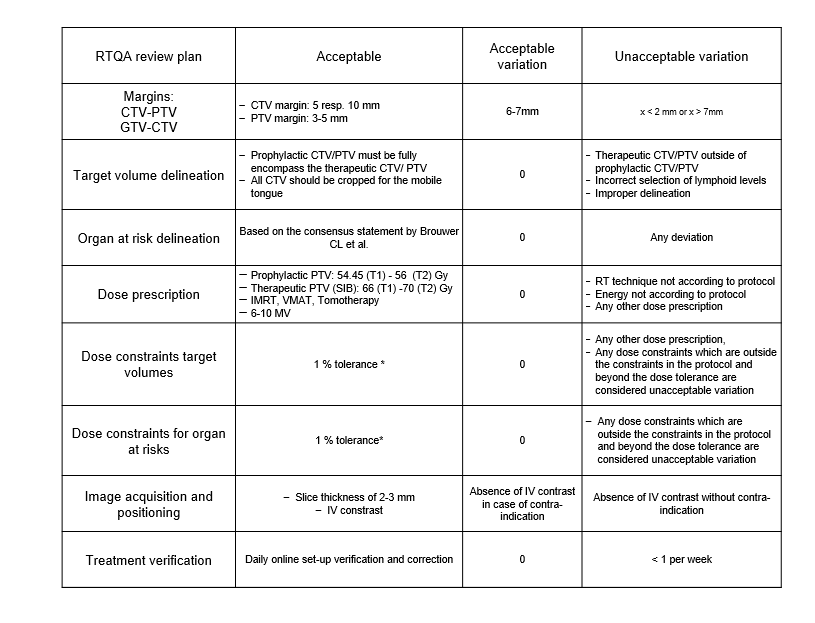


**Supplementary table 1:** Predefined criterions for BC review.

| **Type of protocol deviations** | **Incidence (n)** |
| --- | --- |
| Prophylactic PTV | 19 |
| Therapeutic PTV | 8 |
| Controlateral submandibular gland | 1 |
| Extended oral cavity minus PTV | 5 |
| Pharyngeal constrictor muscles | 8 |
| Glottis and supraglottic larynx | 5 |
| Mandibule | 6 |
| Brainstem PRV | 3 |
| Spinalcord PRV | 3 |
| Cricopharyngeal inlet | 5 |
| Cervical esophagus | 4 |
| Dose thyroid | 3 |
| Dose prophylactic PTV | 2 |
| Dose glottis | 1 |
| Dose ipsilateral parotid | 1 |
| Dose mandible | 2 |
| **Total:** | **76** |

**Supplementary table 2:** Description of all unacceptable deviations for all of the initial submissions
